# 2D Fast Vessel Visualization Using a Vessel Wall Mask Guiding Fine Vessel Detection

**DOI:** 10.1155/2010/580518

**Published:** 2010-07-29

**Authors:** Sotirios Raptis, Dimitris Koutsouris

**Affiliations:** Biomedical Engineering Laboratory, School of Electrical & Computer Engineering, National Technical University of Athens, 9 Iroon Polytechniou Str., H/Y Building-Zografou Campus, 15773 Athens, Greece

## Abstract

The paper addresses the fine retinal-vessel's detection issue that is faced in diagnostic applications and aims at assisting in better recognizing fine vessel anomalies in 2D. Our innovation relies in separating key visual features vessels exhibit in order to make the diagnosis of eventual retinopathologies easier to detect. This allows focusing on vessel segments which present fine changes detectable at different sampling scales. We advocate that these changes can be addressed as subsequent stages of the same vessel detection procedure. We first carry out an initial estimate of the basic vessel-wall's network, define the main wall-body, and then try to approach the ridges and branches of the vasculature's using fine detection. Fine vessel screening looks into local structural inconsistencies in vessels properties, into noise, or into not expected intensity variations observed inside pre-known vessel-body areas. The vessels are first modelled sufficiently but not precisely by their walls with a tubular model-structure that is the result of an initial segmentation. This provides a chart of likely *Vessel Wall Pixels* (VWPs) yielding a form of a likelihood vessel map mainly based on gradient filter's intensity and spatial arrangement parameters (e.g., linear consistency). Specific vessel parameters (centerline, width, location, fall-away rate, main orientation) are post-computed by convolving the image with a set of pre-tuned spatial filters called *Matched Filters* (MFs). These are easily computed as Gaussian-like 2D forms that use a limited range sub-optimal parameters adjusted to the dominant vessel characteristics obtained by Spatial Grey Level Difference statistics limiting the range of search into vessel widths of 16, 32, and 64 pixels. Sparse pixels are effectively eliminated by applying a limited range Hough Transform (HT) or region growing. Major benefits are limiting the range of parameters, reducing the search-space for post-convolution to only masked regions, representing almost 2% of the 2D volume, good speed versus accuracy/time trade-off. Results show the potentials of our approach in terms of time for detection ROC analysis and accuracy of vessel pixel (VP) detection.

## 1. Introduction

Living beings can survive and operate when the functioning of their body's vessel network remains unhampered and thus when blood circulates normally through their vessels and arteries. Therefore, vessel detection is an indispensable diagnosing tool to monitor the situation of the body's blood traffic. This puts vessel detection diagnosis systems at the core of a large variety of applications in medical *Computer Aided/Automated Diagnosis *(CAD). The vessels throughput, good operability, and maintenance in a healthy condition is the issue in most medical operations from the 1st aid to complex surgical in-hospital operations. 

The work presented in this paper puts the emphasis on pixel modeling applications for the retina tissue of the eye. Pixel modeling consists of a two-stage approach that first facilitates visualization and then improves the detection of the fine structure for eye vessels. Pathologies that are eye retina-specific can be related to small retina structure. Even subtle changes on the peripheral network of the vessels can be related to a range of diseases such as diabetes, hypertension, stenosis, and atheroscleroma. They all refer to changes on features of vessels and can have direct geometrical interpretation. Vessel early endings or cross-section changes, abnormal cuts or even vessel proliferation (*angiogenesis*) seem to be related to at least an equal number of pathologies or not healthy conditions, and then more attention is required. This can be contrasted to having an overall picture of a normal vessel network for differential diagnosis purposes. Indeed, most of the above fine changes do not alter the vasculature as a whole and are actually hardly observable if not sufficient information has been made available for local in depth inspection. This difficulty makes early diagnosis a very challenging task. 

More specifically, the challenge faced is in designing successful vessel detection systems and algorithms to capture faint and not easily seen changes over long periods of time. Such changes refer to the characteristics of the small scale vessel structure. The observation scale is very important. The size or the range of changes can be as small as a few pixels decrease in a vessel's diameter (*stenosis*) to the presence of some additional tens of pixels in a vessel's cross-sections (*thrombosis*). The scale itself needs to be carefully detected with a family of techniques. scale gives an insight into the expected spatial extent of the local phenomena. Also, the scale dictates the range of variations that can be met. To study the changes in the vasculature, there are three major strategies followed in the literature. In the first strategy the system detects tubular or pipe-like structures on retinal images by exhaustive convolutions with a set of *Matched Filters *(MFs). These would differ in scale, orientation, magnitude, spatial sensitivity, and zero crossing frequency, to cite the major parameters to tune in MF's [1]. The second family accommodates approaches that perform 2D curve tracking given a selection of set of initial points (seed points) and are first carried out. Pixels with more salient vessel properties are first defined manually and, in the sequel, intermediate level pixels satisfying vesselness properties are inferred. The major criterion is these pixels lying at acceptable positions along the tracking path that seed pixels designate. They are then incorporated into the main body of the vessel's skeleton. Still, these pixels are often likely vessel-wall pixels, and as in [2]. Vessel final shapes can be defined also by using landmark pixels also known as forming “snakes”. The approaches that use or compute data-driven shapes mostly fall into the category of *active contour *approaches for vessel detection. In vessel ridge detection approaches, after skeleton pixels have been defined then the algorithm needs to find walls and the rest of the inside-vessel pixels. Doing this requires prior knowledge. The width sought or perhaps the cross section's profile can guide local search. Then border pixels are used as landmark points. The initial pixels for vessel segmentation can either be wall pixels, which play the role of starting points for finding the entire vessel network, or it might also be center-line (CL) pixels. This depends on what data can be more easily available. Many vessel detection schemas follow the (CL) approach as the first task to do [3]. A vessel's CL is easier to detect and has more salient features such as the high curvature and being at the center of symmetrical edge information, that is, between high transition and edge vessel sides-endings. The local variation then leads to compute the width of a vessel and decide whether, in principle, there is a change in basic vessel parameters. 

The method presented in this paper falls most likely into this category. Still, it requires no spotting manually points. It comprises a module that yields a precomputed basic vessel structure as with the VWPs. The rest of vessel pixels (VPs) that are not walls are filled in locally. The method proposed differs in that a mask is easily computed and not manual operation is performed. Then the MF's operate as guided by the mask. The VWPs define likely borders and local properties fine-tune the MF for finer segmentation of the rest of the vessel's body. This saves not only tedious manual operations but can also save time from applying extensive MF convolutions to regions that are not predominantly characterized by vesselness.

Vessel detection for visualization needs to be contrasted to detailed, diagnostic vessel detection. When depicting the range of the network, it is often enough to model the main body, and approximate the size and locus at bifurcation points or at end points. This covers most of the branching of the vessels. Main vessel-body detection can be effected using less parameters such as length, orientation, and width and at fewer sampling points. However, diagnostic-modelling techniques may dramatically fail when the geometrical parameters computed are not reliable enough to screen precisely the small details of the peripheral network. It is quite often the case that one observes noncontinuous vesselness properties, texture noise, or not expected vessel intensity variation inside the vessel body area. Diagnostic vessel detection needs not to establish a model for the entire network but it works on fine details or long-time changes in the structure of it. These might be very important for alerting of early retina related pathologies. Fine vessel detection as a diagnostic technique can offer great insight when used with other vessel types such as heart, brain, or other human organs dependent on vessels' proper condition. For purposes of differential diagnosis, one would need the same main network in many instances in time and update about changes occurring as a result of pathologies incurred in the meanwhile.

The role of VWPs and of the VPs that are not VWPs is an innovative concept in this paper. Central to the concept of vessels is the definition and mathematical approximation of border pixels that belong to the frontiers of a vessel's tissue. In classical image processing a kernel idea is that to define a body/object on the 2D image plane one would need well-sketched edges that can make up continuous edge segments, that is, line-segments. Well-linked smooth or not too erratic line segments can build borders. Borders confine groups of pixels that are spatially connected and possess similar properties. These pixels are then thought of as belonging to compact feature-homogenous regions called segments. Segments often cannot be strictly filled in with same property pixels. The criterion is that the vast majority of the pixels in a single segment coincide, in their basic properties, depending on the sensitivity of the application at hand. The rest of a segment's locations can be filled in with pixels that differ from that segment's average properties. Still, this should no exceed an inclusion's tolerance threshold. The tolerance can be measured in a number of Gaussian sigmas or standard deviations from the mean value for this property. Then these in-segment outliers complete the segment's body so as to obtain plain region without holes or discontinuities. In further computations sparse pixels, initially not fulfilling basic segment's properties, can be assumed as parts of the segment. Certainly, this is mainly due to their close spatial proximity to that segment's prototype pixels and driven by the need to have segments as compact as possible. Although having absolutely homogenous and as compact as possible segments is best for good visualization it is not necessary for mathematical treatment. Clusters or segments only need to be manipulated as arrays of pixel-wise mathematical descriptions that have consistent properties. 

The role of VWPs and VPs in this context given above is obvious. We need well-separated VPs bordered by VWPs. Nevertheless, imperfect VPs can be tolerable so long as they add to our having a useful vessel/nonvessel spatial pixel segmentation. We may have VPs that are not wall pixels and have gradient or intensity or spatial derivative properties away from the acceptable values. If these pixels are found in a VP relatively homogenous region and are bounded by good VWPs then they are also accepted in this segment. Hence, the mask provided by the VWPs aims at gathering such outliers. We end up with segments without inner gaps or holes. 

The previous analysis stresses the fact that border pixels need to be very successfully detected as they will lead a segment's recruitment process. In this paper we therefore put an emphasis on the most characteristic vessel border pixels, which are its wall pixels.

Nevertheless, we need to face the problem of false vessel border pixels and also find a reliable solution for the spatial relationship between walls and vessel body pixels. Correct VWPs essentially assist the diagnostic process. Also minimize severe initial segmentation errors. Such could be, for example, beginning a fine segmentation and a detailed region filling when poor borders have been detected. We thus employ the notion of a chart of likely VWPs. A chart when contains only two types of regions, that is, vessel wall pixels and non vessel-wall pixels is often called a binary map or a mask. We discuss a method that first builds up a map. There is an obvious mutual relationship between VWPs and VPs as discussed above. In vessel detection, we make it more clear. Vessel walls help define the VPs by spatially appropriately juxtaposing/pairing VWPs and then by spatial interpolation. The vessel properties used to define vessel interior pixels, that is, the VPs that are not VWPs, are not the same for these cases. The interior VPs need to follow the cross-section patterns. Vessel interior pixels mostly serve, in this paper, pictorial purposes, as it will be clear in the results section. Walls need to roughly separate vessel tissue pixels from nonvessel tissue pixels. In relevant works, the emphasis that is placed here on border pixels or on wall pixels has been put on *center-line* (CL) or skeleton pixels detection. CL pixels and VWPs are interchangeably required to fulfill end point patterns. End points patterns are very critical in diagnostic vessel detection. The transition patterns refer to gray level (GL) variations when getting from a near-to-wall but within-vessel region to an outside-of-the-wall or background or soft tissue areas found. These are usually in the close vicinity of the vessels. 

Inversely, when interior VPs are first or roughly defined as an entire network then the VWPs that were first used to make the caricature of the network are better assessed and located. So, VPs can help better finding back VWPs. This is done with much less effort as would be needed when applying extensive and expensive 2D convolutions spanning over much wider scale ranges. The well-known *Maximum Intensity Projection* (MIP) method actually iterates over successive 2D convolutions over a continuous scale spectrum and finds maximum projections across different orientations and scales. We limit these iterations to a few candidate scales using the local texture features used when designing the mask. Indeed, this is accomplished by looking to more global properties like texture, not available before since much less pixels were known to belong to the vessel tissue. Masks can help with visualization since the basic structure is already computed. 

In the relevant literature the most commonly referred to method to find vessels is by easily tuning a limited set of MF's. The gradient information provides insight on large GL differences. When the retinal image is first smoothed using a generic Gaussian filter then the GL transitions and gradient information become more evident. Sporadic edges or vessel-like structures give large local gradients as well but statistically only a very small portion of them belong to walls. This could hamper a good detection. The solution to this is VWP pairing. To determine wall characteristics the Gaussian image's gradient and the gradient's spatial arrangement parameters are examined. Spatial restrictions were applied to high edge or gradient pixels and kept the cost for the detection quite low. This is quite important considering the fact that for a full vessel detection one usually needs to go through a number or different scales and orientations that represent the vessel's expected scales and likely orientations over the entire image space. 

The paper is organized as follows: in Section 1, we provide an outlook of how likely VWPs can be defined using a gradient and local spatial coherence criteria. Furthermore, the notion of wall pixels is studied in the context of edge information. As an extension, the basic formulas for gradient, orientation, and curvature information and their variance are explained. A note on local homogeneity is also discussed. A more detailed presentation of the texture descriptions used in the vasculature map is given in Section 2. We introduce features that account for local spatial periodicities as the *Spatial Grey Level Difference Statistics*. A detailed discussion of the transition from a map of wall pixels to vessel pixels is also given. In Section 3, we discuss how MF's can be adjusted using features computed locally. For that we use highly tunable filters that can be adjusted to many profile patterns and also discuss how transition models can be incorporated into this framework. Finally, detailed still representative results are provided in Section 4 for three vasculature cases and all detection stages are depicted along with comparative illustrations using ROC analysis for full scan and wall map guided scans. A indicative table is also with time comparative performance in Table 1.

## 2. Vessel Wall Detection

### 2.1. Wall Detection and Edge Alignment

Rough VW detection is rather a straightforward and easy to implement technique in its greater part. The relevant literature is rich in methods that begin from finding the walls and then extract the entire vessel structure. In [4], the Can algorithm is presented that uses different wall finishing patterns to extract walls. Walls are extracted as groups of strong edges aligned along parallel and antiparallel lines. As opposed to that, VWPs' very accurate detection is not a trivial task since wall edge pixels are abundant in retinal image. Reasoning is applied to locate aligned edges that coincide with locally maximal gradient pixels. Alignment is assisted by the adoption of a grid over the retinal image. The wall pixels cannot be usually deterministically defined in their very details. These are though needed in the diagnosis of eye-diseases. Such details can be enhanced by applying small range contrast features that are computed over a set of neighboring blocks around examined locations. A weighted policy can then balance their contribution to a block's center-pixel being a wall pixel. This is proposed in [5], where the so called the Weighted Local Variance feature is introduced. The weighting is done by a Gaussian kernel in order to account for the relative orientation and thus the contributions of the neighboring blocks. This can further assist the detection or local curvature changes, which are essential to the successful tracking of vessels. 

Edges and gradient pixels used in primary vessel detection need to be filtered by a threshold function in order to decide on further consideration and thus inclusion into the rest of the vessel body. The choice of an adaptive value for the threshold is an intriguing point in this process. A way to insure adaptation to the local image is to take block histograms and study how contrast, edge or gradient variation characteristics change as blocks are being scanned by the detection algorithm. These variations are manifested and best modeled as distributions. Interesting block centers that are selected are then assumed to likely be VWPs and the distance and alignment between them is kept for further consideration. The aim is to spot such candidate pixels in spatial arrangements that minimize distances, respecting restrictions posed by alignment. These restrictions usually concern local block orientations. Empirical algorithms are used to put in a reasonable spatial sequence the VWPs. This is as an alternative to more formal optimization schemas that are employed when we examine specific geometrical topologies that might fit the data. For example, algorithms for computing “snake” models for 2D curves for vessel borders can be such. Empirical algorithms can be useful when the edge/gradient information for the optimization be efficiently applied is not organized or enough. Another reason is even when there are missing data from not continuous edge tracking. To this end, a known algorithm is the *Sequential Linking Algorithm *(SLA) discussed in [6]. The parameter used is the tolerance to the local curvature. Finally, morphological techniques may be used to eliminate or to complement the VWPs detected by other methods. Thus, vessel boundaries can be smoothed or corrected by exclusion or inclusion of pixels on at their limits. This is also discussed in [7].

In the method presented here, we define a map of likely VWPs using easy to compute gradient-based features for a start. The gradient features must be suitable in orientation, distance, magnitude, and concentration. We need to distinguish vessel boundaries from noninteresting, lengthy edge segments or from vessel-end like structures. The retina contains many uninteresting various sizes bodies with cross-sections that may confuse detection. There are a number of retina formations that can trap algorithms but do not belong to the vasculature sought. To overcome this, we define a set of intensity profiles near the likely VWPs. Normally, we can expect single light-dark or dark-like smooth transitions to be centered around VWPs. Such an algorithm could be applied as an additional fine wall segmentation step and does not model the vessel itself. A typical semi-, or uni-modal Gaussian or sigmoid step function can approximate this variation across the wall. Reported in the literature are variation patterns that exhibit a two Gaussian modes profile. In this case the wall serves as a natural frontier between two relatively homogenous areas or limited range and different average GL's. This would be the outer darker and the inner somewhat lighter or the inverse. Although not very often met, a two-mode Gaussian with differently valued modes (peaks) can give us an insight on whether this phenomenon takes place. Once we define a map, then this map most probably contains lots or sparse pixels that have nothing to do with any part of the vessel we are looking for. We then need to apply a set of MF's well-tuned in scale, orientation and spatial variation as dictated by the original image's features and at pixel locations defined by our map. The distance parameter can be used as well. This parameter can be computed with reference to the closest wall pixel location that separates candidate VPs from its paired likely wall pixel. Distance can provide an estimate of the scale, orientation and spatial variation to adjust the MF to. The benefit is that we do not need to apply the MF over all retinal pixels. After detecting the walls the CL of the vessel can be tracked by the roughly or perfectly paired boundary pixels. The application of a highly tuned MF can be guided by the CL as well. When two VWPs cannot be exactly paired then it is relatively easy to detect such discontinuity and interpolate from other well-paired pixels. To the extent to which this loss of tracking does not happen over a large area the interpolation can make-up for the lost paired pixels. A guide for that is the local gradient features computed at nearby good map locations. After the application of the MF's we follow the standard path and take the maximum matched filter response (MFR) to correct the angle and width roughly detected when using the paired pixels. It may occur though that the parameters thus computed give contradictory results and give not continuously observed track points. Or it may happen as well that the walls distance as computed by the MF does not agree with the parameters originally computed from the paired VWPs. Then, either there is no actual vessel at the point the MF was initially applied or there is a change that cannot be captured. This can occur at points with very high curvature, high tortuosity, or with frequency noise. A frequency noise is when the initial spatial Fourier frequencies are captured wrongly. The algorithm then stops and manual delineation of the local vasculature is carried out.

### 2.2. Vessel Wall Pixel Maps

The *Vessel Wall Pixel *maps (VWPs) are 2D binary fields that help roughly locating vessel structures or spotting vessel pixels as mentioned. The exact vasculature can be then obtained by fine-filtering the region around a VWP using preselected MF's with local features that are sought structures specific. The result is a likely *Vessel Pixel *(VP) map or a *Vessel Map* (VM) of pixels originally. These are assigned a feature vector that contains descriptions related to the local dominant phenomena.

In the current literature as in [7, 8], VM's are not conceived as an intermediate stage for vessel pixel detection. Instead, an anisotropic and orientation-specific filter is applied to detect dominant orientation and a statistical cost-function is applied to extract pixels that that are deemed as true VPs. 

In this work, we adopt a computationally lighter approach using local features very convenient and fast to compute. These features include orientation ${\hat{ο}}_{ij}$ at some seed-pixel location, (*i*, *j*) the degree of its dominance *s*_*o*_*ij*__ = *s*_*ij*_, which gives the intensity of the feature, scope information *r*_*o*_*ij*__ = *r*_*ij*_ which is related to the spatial extent to which this feature is valid and describes local phenomena, the main texture *t*_*o*_*ij*__ = *t*_*ij*_ which provides local information on how local gray-levels (GL's) change within the neighbourhood or how often GL differences are observed locally. The exact definition of these features is given below.

### 2.3. Local Orientation Features

The orientation information can be traditionally measured using the direction unity vector that is vertical to gradient field of the image GL or elevation map, ∇*I*(*x*, *y*), for a retinal image *I*. The gradient direction is vertical to the vessel's orientation. That is: $\langle {\hat{ο}}_{ij},\overset{̅}{\nabla }I_{ij}\rangle =0\wedge \left|{\hat{ο}}_{ij}\right|>0$. Vessels are usually micro-tube-like structures. They are thus elongated structures with low local or inner variance in their inside characteristics and proclivity to higher gradient towards the vessel walls or boundaries. They present arbitrary but well observed orientation. The vast majority of one vessel-segment's pixels are strongly homogenous in orientation. Hence, we adopt a local reference for orientation with the boundary pixels orientations being in parallel or in anti-parallel direction relative to the single local orientation sought. Let us designate a local coordinate system as $\hat{u}$ for the direction along the vessel's CL and the direction crossing this as the second major orientation, $\hat{v}$ which is parallel or anti-parallel to the local gradient, that is

(1) $$\langle {\hat{v}}_{ij},\overset{̅}{\nabla }I_{ij,n}\rangle \in \left\{-1,1\right\},\overset{̅}{\nabla }I_{ij,n}=\frac{\overset{̅}{\nabla }I_{ij}}{\left|\overset{̅}{\nabla }I_{ij}\right|}.$$

The $\hat{u}$ direction is supposed to have a very low gradient for VWPs and VPs and high $\hat{v}$ as it is vessel end point. All examined VWPs as location indexed as (*i*, *j*) depending on their positions on the retinal image we denote it as ${\hat{v}}_{ij}$ or ${\hat{u}}_{ij}$ accordingly. The challenge is to decide on whether we can have a single or locally average $\hat{u}$ for all pixels around a seed pixel (*i*,*j*)_*c*_, which then gives the local dominant orientation. The strength of the orientation is given by its relative gradient-strength measure that accounts for the absolute magnitude of the orientation's gradient vector field with respect to the average orientation gradient field. That is

(2) $$\begin{array}{cc} s_{ij} & =s\left(\left|\nabla I\left(i,j\right)\right|\right)\\ & =GS\left(\left|\nabla I\left(i,j\right)\right|\right)\\ & =A\exp\;\left(-\frac{{||\nabla I\left(i,j\right)||}^{2}}{\left(2*{\sigma_{\nabla I}}^{2}\right)}\right), \end{array}$$

where *σ*_∇*Ι*_ = 1/(*M* × *N*)∑_*i*_^*M*^∑_*j*_^*N*^(||∇*I*(*i*,*j*)||−||∇*I*_*M*_||)^2^ and ∇*I*_*M*_ = 1/(*M* × *N*)∑_*i*_^*M*^∑_*j*_^*N*^|∇*I*(*i*, *j*)|.

Where *A* is chosen to be a locally sensitive parameter

(3) $$A=\underset{\left(i,j\right)\in N_{ij}}{\max\;}\left\{\nabla I\left(i,j\right)\right\},$$

for some neighborhood around an examined point (*i*, *j*), while *I* = [*i*,*j*]_*i*,*j*=11_^*M*,*N*^ is the set of pixel coordinates. The scope of the orientation is related to the homogeneity of the local gradient field. A vessel pixel must be surrounded by a region of pixels with quasi-constant orientation. For that we are assisted by the standard deviation of the gradient on both directions $\hat{u}$ and $\hat{v}$. Still, a Gaussian measure is used that suggests the radius of the area as the scope of the local orientation: *r*_*ij*_ = *r* : ∃*G*(*σ*_∇*I*_(*i*, *j*), *t*(*r*, GLD)) ≥ thr_*σ*_∇*I*__. The argument *t*(*r*, GLD) is a texture descriptor that describes an image where the average Euclidean distance between two pixels with the same GL is *r* while the average GL difference between connected pixels is GLD grey-levels. Connected pixels are those pairs of pixels ${\overset{̅}{p}}_{1}$,${\overset{̅}{p}}_{2}$ for which: ${\left[{\left({\overset{̅}{p}}_{1}-{\overset{̅}{p}}_{2}\right)}^{T}\left({\overset{̅}{p}}_{1}-{\overset{̅}{p}}_{2}\right)\right]}^{1/2}\in \left[1,1\sqrt{2}\right]$ where ${\overset{̅}{p}}_{j}={\left(x_{j},y_{j}\right)}^{T}$, *σ*_∇*I*_ is the standard deviation of the gradient field at location (*i*, *j*) where the area of averaging is defined by a minimal-sized 8 × 8 block.

## 3. The Spatial Grey Level Statistics Metrics

Local texture content can help defining critical parameters used in vessel segmentation. Wall pixels can be more easily discriminated from the complex retina background. Wall and CL or ridge pixels often resemble to diffused but not useful textured pixels that might confuse the application of the MF's. As a generic concept texture information is very critical especially in medical imaging. In many applications that deal with complex and texture-rich images, the information that one might think of as a useless background can be turned out to be the critical one. In fact, many pathologies in medical images, like the diffused ones (attacking the liver or the kidneys), appear in the form of characteristic textures. These textures must be separated and studied carefully [9]. 

Texture is constructed from small variations of the GL values. These are usually accompanied by a spatial periodicity. By that it is meant that short-range intensity variations may be modelled or approximated by studying image differences when the image is shifted by a certain vector quantity. The later is often called the *displacement vector*. Then, the eventual periodicity may be revealed by simple GL matching. In vessel detection, the main periodicity we attempt to find is the symmetry around the CL of a vessel. Furthermore, it is interesting to study GL spatial relationships and construct such relations by means of cooccurrence probabilities. When pixels with large differences happen to appear at short distances from each other, then the texture for this image might be characterized as complex. When such differences are observed rather at long distances, then the texture seems to be rather soft. Large variations in homogeneous areas are likely to occur at equally large distances. When detecting structures, we assume the target is to find homogeneities as contrasted to borders' sharp changes that separate structures. Such observations can be mathematically formulated under the matrix form of cooccurring GL events. As a GL event can be taken a GL observed at a certain planar or a 3D position. A combined GL event is when possible GL values are combined, pair-wise, to see which ones occur most often and how are they spatially distributed. In this case, a displacement vector that connects them is part of the formulation. 

Even when using this kind of techniques it is still impossible to characterize texture properties exactly. To extract major differences between them is a possibility we employ in this work. It is rather very difficult to mimic mathematically the human eye and define such features. To this end we use a major texture quantifier, the *Spatial Gray Level Difference *matrix also known as the SGLD matrix. 

S.G.L.D. matrix can be used to derive texture features. It provides in tabular form information about basic spatial statistical properties for texture. It is often used in distinguishing *Regions Of Interest *(R.O.I.'s) from normal tissue in medical images. A displacement vector $\overset{̅}{D}={\left(d_{1},d_{2}\right)}^{T}$ is first defined over the image plane. The (*k*_1_, *k*_2_)-th element of the S.G.L.D. matrix is defined as the joint probability that the GL's *k*_1_ and *k*_2_, with *k*_*i*_ ∈ [0,255] occur at a distance of $\overset{̅}{D}$, $p_{\overset{̅}{D}}\left(k_{1},k_{2}\right)$. That is

(4) $$\begin{array}{cc} \text{S}.\text{G}.\text{L}.\text{D}._{\overset{̅}{D}} & =\left[\begin{array}{cccc} p_{11} & p_{21} & \cdots & p_{N1}\\ p_{21} & p_{22} & \cdots & p_{2N}\\ \vdots & \vdots & \vdots & \vdots \\ p_{N1} & p_{N2} & \cdots & p_{NN} \end{array}\right],\\ & \;\;\;\;\;\;\;N=256. \end{array}$$

The S.G.L.D. features are affected by the image's depth because of the trade-off between the GLs' resolution (number of GL's per number of binary digits) and the statistics of the estimated joint probability distribution. If the image's depth is high, then the joint probability will be poor. If the depth is low, some characteristic features of the distribution will be lost. The S.G.L.D. features mainly reflect the distribution of the elements in the S.G.L.D. matrix such as mean observed distance, energy, correlation, variance and are information theoretic. A survey can be consulted in [8]

When practically applying S.G.L.D. features on a vessel image there is some prior knowledge we can use to avoid manual operation. All $\overset{̅}{D}$'s are not examined. We are specifically interested in likely distances separating paired wall pixels. This is easily known from the research community in the field. We tried displacement vector magnitude values within the interval $\overset{̅}{D}\in \left[2,30\right]$ in pixels along both direction –x and –y. The orientation was approximately known from the wall pixel map. For strong and paired wall pixels we only had a limited set of distances that could separate them. The angle computed was the angle we rotated the vector $\overset{̅}{D}$ by. The number of local iterations over likely angles and vector magnitudes is significantly lower than the full range [0,*π*]. Also, the area of the solicitation region is bounded. Then, we only needed to carry out very focused MF 2D convolutions. Also we ended up being more precise than running a full MIP process as it would be the case if we had to explore all possibilities over the entire image plane. *Τ*his was also manifested in the ROC curves presented in the Section 4 where some results are shown.

As already mentioned, the local search might yield a limited but still large number of successful high edge/wall pixel-pairs that are still not vessel border pixels. The local homogeneity criterion applied was the local variance for the gradient and for the wall pixel curvature features. This further limited our options. This was good because local homogeneity criteria acted like spatial filters. Finally, nonvessel wall pixels that escaped that level of restrictions were eliminated using a simple sporadic pixel brush like a 4-connected region growing pixel algorithm that at almost no cost removes them.

## 4. Fine Vessel Segmentation Using Matched Filters

### 4.1. How Can a VWP Define Basic MF Characteristics

In the previous sections, we saw that wall pixels define a map, which can reduce the research space for a vessel detection application. After obtaining a working map pointing where most probable wall pixels might be found we can begin our local search of best VPs. Vessel walls can be then fine processed as groups of VWPs of high probability. Gradient, scale, orientation and their variance as well as good pairing are used as arguments to in a Gaussian similarity kernel. The similarity kernel is adapted for the local images. That means that we adopt a global set of descriptions but the statistical means and variances or other features involved are local. It is in a multivariable Gaussian kernel. The Gaussian grades thus local behaviour with respect to the wider area. Hence, true VWPs need to pass this criterion. We then apply a limited bank of MF's on best VWP points. MF's have almost preselected scales and orientations and are adapted to the local characteristics. The role of the MF's is to enhance true VPs and accord a low membership to outliers. This is the fine-segmentation process about. MF's are aimed at finding, adjusting, and more accurately computing small details not captured by the VWP map or by the gradient only based descriptions. On the other hand, equal part of the refinement process is to eliminate remaining false alarms of less significance. Such would be noise, small drusen, crossing vessels perpendicular to the observation level, or other structures. 

Fine vessel diameter or width and shape changes are the ultimate target of a retinopathy computational diagnostic schema. This is a fact since most of the early changes in the retinal vasculature are very hard to early observing even by the most experienced ophthalmologists. 

The VWP map provides a set of candidate scale and diameter values for the region examined which are fed into the MF as its parameters. The pairing information provides insight into the spatial limits for the MFs' 2D convolutions. The level of the relative MF responses (MFRs) after convolution as well as the spatial variance of the high triggering, seed points detected lead the final decision on whether a pixel is a vessel pixel (VP) or not. 

The interior VPs can be inferred as pixels between reliable VWPs by applying a simple interpolation algorithm on VWPs' MFRs. Bad/low MFRs at good VWPs' points might develop discontinuities. Bad MFRs are pixels that are close to true wall points but give MFRs out of an acceptable range. Acceptable MFR ranges are conceived in a way to confirm the basic geometrical properties a vessel's cross-section profile is supposed to have. For example we cannot accept a considerable fraction of pixels lying along the line that connects two well paired VWPs having dark GL's or GL's that have are different sign from the sign most aligned pixels have. The fraction depends on the noise level in the area and on the vessel width observed using the texture's SGLD measures. The vessel width can only be precisely detected when two candidate VWPs that are reliably paired, and most of the VPs found in between them are high MFR points. For well-paired VWPs but with high MFR variance we finally observe sparseness/diffusion. This means we have isolated VWPs, which cannot be connected by well-aligned VPs because the VPs give very different MFRs. If this happens to a locally large extent (wider than the local search area), then we cannot approximate suitable MFR points by the closest well-connected and well-paired VPs or by the VWPs. Then, these not segmented pixels are put out of the list used to build up the local vessel structure. In this problematic case all local vessel parameters need to be re-evaluated. However, vessel recovery is another field of study and is beyond to the scope of the current work. It pertains, as a topic, to the paradigm of recovering damaged primary information. When encountered, this case, it was treated as missing information or simply a false alarm/noise case. 

The measure for high MFR sparseness, as mentioned, is a dynamically tuned Gaussian kernel adjusted to put away pixels with a single (i.e., not well connected) but high MFR. 

Suppose we encounter a wall pixel, as *i*-th in the local list with probability, ${\overset{̅}{p}}_{WP,i}$, for being a good VWP that has an acceptable MFR. Then, one of its candidate paired pixels denoted as ${\overset{̅}{p}}_{WP,i}^{\prime }$ has high MFR and an acceptable SGLD distance from the first. Then, the second pixel ${\overset{̅}{p}}^{\prime }$ is not suitable for pairing (with ${\overset{̅}{p}}_{WP,I}$) when there are not enough neighboring pixels, to the paired one, with similar properties to it. That is, the neighbors of the 2nd pixel must be good VWPs and the 2nd pixel's VWPs similarity must fall within a region of empirically less than 2 sigmas or standard deviations from the closest well paired VWP. This corresponds to an MFR of roughly 1/(2*e*) from the closest best MFR. Approximately the same analogy holds for the Gaussians describing the relationship of the gradient and orientation and curvature information. All properties need to be relatively homogenous. When this restriction is not satisfied for both the good 1st VWP and best pair, the 2nd is not kept. Any eventually connected VPs are re-assigned to neighboring VWP pairs. 

It is obvious that such a detailed local criterion forces homogeneity, ensures symmetry, and is good at examining small changes occurring in time on the local vessel structure. That saves large amounts of convolution time. Indeed, there is no large-scale search and we do not spend time with many different orientations. This is though compromised by severe local search when there is a need for one. It is probable, though, that good and well-paired VWPs are not actually the best ones. Then, centering the inclusion Gaussian criterion around their features might end up with some loss of information. Similar properties can be met in cross-section like forms as well. Our argument here is that if there were not enough good primary information then any vessel detection schema would fatally fail. The tolerance of the Gaussian *σ* for all features considered depends on the local deviation of the neighboring good VWPs' similarities, for all features *F*_*j*_, *j* ∈ [1,4],

(5) $${\overset{̅}{p}}_{WP}=GS_{F_{j}}\left({\overset{̅}{p}}_{\text{cand}}\right)=\exp\;\left(-\frac{{\left[E_{F_{j}}\left({\overset{̅}{p}}_{\text{cand}}\right)-\mu_{E_{F_{j}}}\right]}^{2}}{2{\sigma^{2}}_{E_{F_{j}}}}\right),$$

where: *μ*_*F*_*j*__ is the average local VWP inclusion feature value for feature *F*_*j*_, where index *j* refers to any of {gradient magnitude, gradient's orientation, GL, contrast}. 

All features are computed very fast and don't consume significant additional computational time. Whatever computational burden is actually spent on computing the Gaussian criterion and to applying the above logical reasoning. This not comparable to the complexity of computing a Gaussian convolution kernel and to the convolution itself as mainstream approaches do.

The MF can be any tunable function. Usually a dual mode Gaussian kernel is defined. A better result is obtained when this function also spatially modulated. This is given in

(6) $$\text{MF}=\{\begin{array}{c} \text{for}u,v\in {\left|u-a_{u}\right|}^{2}+{\left|v-a_{v}\right|}^{2}\leq D,\\ =\frac{1}{2\pi {\left(\sigma_{u}\sigma_{\omega }\right)}^{3/2}}\left(u^{2}-{a_{u}}^{2}\right)\left(v^{2}-{a_{v}}^{2}\right)\\ \;\times \exp\;\left(-\left(\frac{{\left(u-\sigma_{u}\right)}^{2}}{2{\sigma_{u}}^{2}}+\frac{{\left(v-\sigma_{v}\right)}^{2}}{2{\sigma_{v}}^{2}}\right)\right)\\ \text{for}u,v\in {\left|u-a_{u}\right|}^{2}+{\left|v-a_{v}\right|}^{2}>D,\\ =0 \end{array}$$

Our MF is a multiparametric, highly adjustable, amplitude modulated, two variable MF suited to capture small variations inside the vessel-body. The critical parameters are: directional local scales (*σ*_*u*_, *σ*_*v*_), the effective convolution kernel size *D* (taken same for both directions), and the spatial modulation parameters (*a*_*u*_, *a*_*v*_) that control the zero crossing points, thus the limits of the kernel.

### 4.2. Basic Algorithmic Schedule: Getting from Coarse to Fine Segmentation

To procure more enlightenment on the essentials of our contribution we are giving a process/data, flow diagram that delineates the information flow among the different modules and the different data forms produced in this process. This is shown in Figure 7 in the Results section. We use a widely accepted *flow-chart* entity alphabet to this end. In this chart, we employ information flow entities represented by directed bold-lined arrows to denote the source and destination of the flow, oval entities to denote processes with labels on them giving a hint on what major data processing is carried out when they are run, rounded rectangles to represent any data produced regardless of the level of the process they are related to. Those are also labelled to indicate the kind of information produced or temporarily stored in the system. The basic concept is the input information that is a raw retinal image as for example, a “tif” file that is part of the world out of the system. Then this information comes for processing into our system and goes through: (a) basic preprocessing that is almost present in all medical imaging systems and includes: smoothing with a Gaussian [8 × 8] kernel or up to [32 × 32] and some rescaling to compensate for illumination nonuniformity and random peak GL-values eventually produced, some filtering that is *per case *necessary depending on the 2D-FFT of the image, and in the sequel the enhanced data are directed simultaneously to two different processes. The first is the computation of gradient field-based descriptions and the second is the definition of a set of sufficiently working displacement vectors in order to analyse the texture further. As shown, we are experimenting with an empirically found appropriate set of vectors producing displaced (*spatially shifted*) images that are statistically processed. Then the co-occurrence matrix is produced that is the kernel module for many texture descriptions. Subsequently, the G.L.D.S. and the S.G.L.D. feature-sets are computed from this matrix and as explained they are computed in a way to account for pixel- and region-wise behaviour when pixels are the center of a suitable region. Texture features permit to approach pixels that can be paired and thus can be considered as candidate VWPs or vessel borders. As shown both paired pixels' distances and gradient features are examined as to their local dominance and homogeneity applying the criteria discussed. Both sets of descriptions are used to better assess the basic characteristics of optimal MF's. Indeed, we are using the tool set of a generic MF bank that uses this feature information to fine-tune specific MF's and thus make them optimally adapted to our images. On the other hand, the initially produced paired pixels guide the construction of a good VWP map. The VWP limits the amount of 2D convolutions. Among the outputs of the basic data flow are the VWP map and the feature-values on locations where VWPs are found.

## 5. Results

Experiments have been carried out with 20 different fundus images taken from the STARE database [10]. We tested the detection algorithm with the VWP map that uses a likely vessel-wall pixel map in comparison to a full scan algorithm using MFRs. Also, tests have been made with other two algorithms, namely the regions growing algorithm and the Center Line based vessel detection as well as with three seed point detection. Numerical results showing the advantages of our algorithm can be insightful in terms of ROC curves and times needed for computing a final vessel map. The results produced are intended to make the point that the time can be reduced significantly and that needless convolutions in regions of unlikely interest are largely avoided. There are still specific performance indicators, which, to the authors' best knowledge are utilized when vessel detection is on focus. They are given in Table 2. They are mainly qualitative measures, though. We projected our algorithm's specifications and/or results to this space of qualitative specifications referred to as method “M6”. Time has not been an explicit concern in the relevant literature so far. Hence, comparative timing data for vessel detection performance are not widely available. We used our own algorithms. Vessel detection accuracy against time for detection is an even more specialized topic. 

Still, timing is an interesting point to look at when detection of vessels is used as an intermediate module for further processing. One example of time-critical and less accuracy-efficient application would be the paradigm of guided surgery. In this area applications exist where a surgeon would need to have a picture of the vasculature before an operation or be roughly guided to the right diseased region when a vessel anomaly is suspected. Then, one would need to locally handle the probe and use more accuracy-intensive methods to exactly spot the point for fine surgery. To this end, of great assistance would be a fine-tuned algorithm or a specific knowledge-based module that uses fast and highly tuned filters in a real time fine vessel detection application. 

Since the topic of fast and accurate eye-vessel detection is a quite specialized one most works in the field tend to fall into a limited range of categories for which few pictorial or specific numerical data are available.

The field is rich though with respect to 3D vessel detection and visualization that is directly applicable to microsurgery applications, still helping surgical guidance. This is actually the next focus of our work.

**Figure 1:**
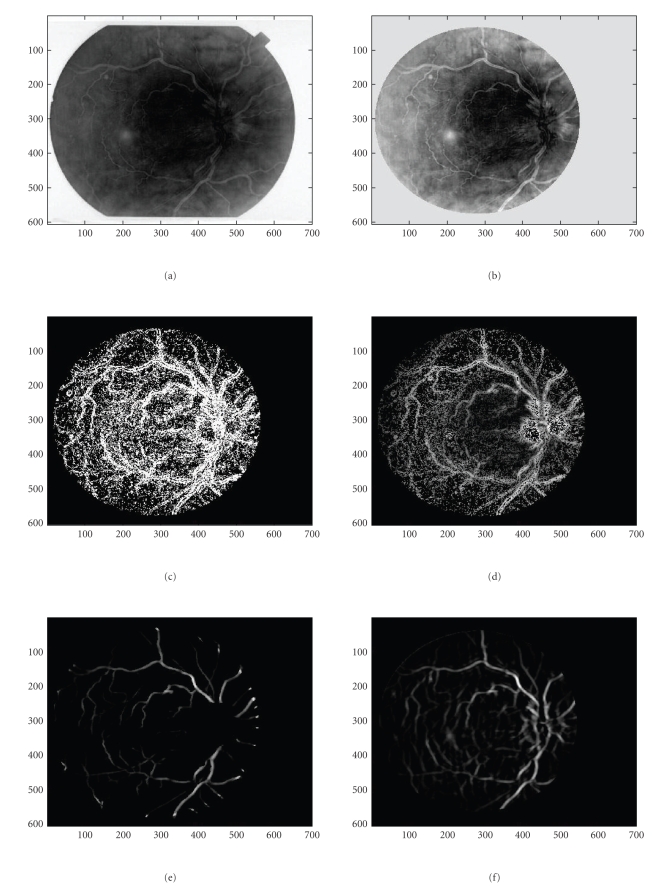

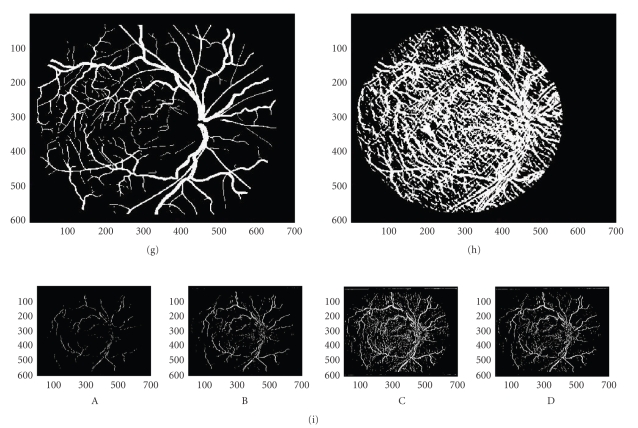
(a) Original fundus image with not well-traced peripheral network. (b) Original fundus image ellipse corrected. (c) Binary vessel wall pixel map (VWP). (d) Vessel map based on VWP. (e) Retinal Vasculature based on VWP and fine segmented for local details. (f) Retinal Vasculature without VWP map using MIP. (g) Ground troth vessel vasculature. (h) Redundant vessel vasculature produced using an Augmented VM and double-sided GS-MG. (i) Successive maps taken using different features and different sensitivity. (A) Gradient homogeneity VMs. (B) Paired VP map and dominant gradient VM. (C) Increased vessel width allowed, lower gradient threshold and interpolated WPs. (D) Experimentally proved VM.

For the sake of performance comparison we give in Figures 1(c) and 1(e) the results using our VWM-based detection algorithm and the manual map produced as in [10]. The manual map is very similar to the detection map produced in the method given in [10]. However, using our structure-sensitive MF as in [1], we have a much finer structure not illustratable neither on the manual map nor in the computer detection map. The obvious but shallow drawback of our method is that we achieve at worse 10% higher number of FP's at the expense of speed. The gold standard map is not as clear though to easily visualize as the small vessel endings or patterns. Still, our result is largely justified by the additional argument that it is better to have more information than less and at better speed as seen in our results for single and dual mode GS-MF. Especially in our case the essential fine and coarse vasculature information is there. This eliminates the need for tedious metaprocessing as would be the case if we had a proven large number of FP's and the ground truth (GT) was in direct incoherence to our detection results. We also argue that the exemplary maps given in the literature are very good but still are estimates of the vessel network. Hence, there is nothing to prove in an absolute and not arguable manner that any possible abnormality can be studied using these GT binary maps. In fact, as it is sufficiently documented and mathematically outlined in [11, 12], we need to combine a capable number of human observers' binary maps to have an acceptably supported GT map as a gold standard. Then we also need to provide the GT results for all logical operations performed in each binary observation map separately. To this we need to apply special statistics as to how one can pool out with confidence experts' binary observations and with which confidence. These binary experts from many relatively and not absolutely reliable observers need to establish a more reliable standard.

In an effort to capture even fainter details, we tested our maps using a more complex and computationally expensive dual-mode [two-peaked], double-sided GS-MF that is not used in [10] which models background/foreground transition pattern (i.e., background/foreground, CL area, and foreground/background), on both vessel walls. Also, the spatial dependence can accommodate zero crossings. These results are given in Figures 1(c) and 1(h) and show more vessel pixels than the results with single mode GS-MF as in Figures 1(c) and 1(g). Some single (Figures 6(b) and 6(e)) or partial double peaked (Figures 6(a), 6(c), 6(d), and 6(f)) are also shown where and can be modeled using spatially modulated kernels in 2D. (Figure 5(b))* show some examples of the dual model kernels for a sampled range of spreads and orientations. These can sufficiently emulate and at low cost the simple wall model individually on either vessel side as a single-sloped wall pattern [dark/light]. The double-sided single mode kernel model is less robust and often cannot capture much information as it assumes a more uniform background that it really is.

The detection of finer details than the conventional methods can achieve, cannot be always observed for all types of vasculatures. The image resolution, on the one hand, as well as the observers' skills and the sensitivity of the ophthalmologists, on the other hand, affect the results and the setting of any gold standard. As we used only online data for this work as in [10, 13] databases we were not able to establish an ideal standard and validate our results in an absolute manner. To our awareness, nevertheless this is still an issue in the concerned community. Consequently, the data we have coped with allow for a limited inspection of the fine details due to the medium resolution. Even quite elaborated and sophisticated filters, as ours would provide the same outcome when the primary rich information is not there. As expected in Figures 1(c) and 1(h), the results can only show tails and a number of sparse pixels. We tried to avoid color-labeled images that most related works are using [11, 12] which illustrate comparative detection for additional features used or hidden pixels/segments they are producing as differently colored regions. For 2D applications that might be confusing. Hence, we elected to use difference binaries for our illustration purposes.

**Figure 2:**
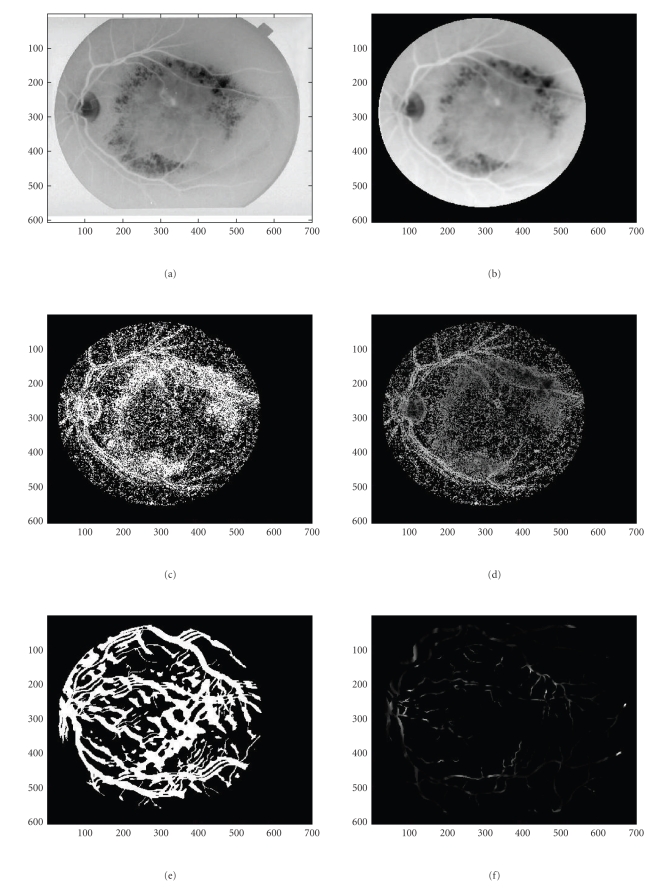

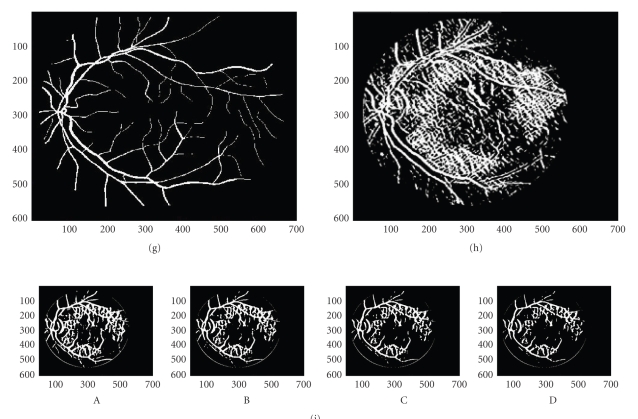
(a) Original fundus image with well traced major network and slightly distinct major peripheral network. (b) Original fundus image ellipse corrected. (c) Binary vessel wall map. (d) Vessel map based on VWP. (e) Retinal vasculature based on VWP and fine-segmented for local details. (f) Retinal vasculature without VWP map using MIP. (g) Estimated ground truth vessel vasculature. (h) Redundant vessel vasculature produced using an augmented VM and double-sided GS-MG. (i) 2nd vessel network: Successive maps taken using different features and different sensitivities (*thresholds*). (A) Gradient homogeneity VMs. (B) Paired VP map and dominant gradient VM. (C) Increased vessel width allowed, lower gradient threshold and interpolated WPs. (D) Experimentally proved VM.

**Figure 3:**
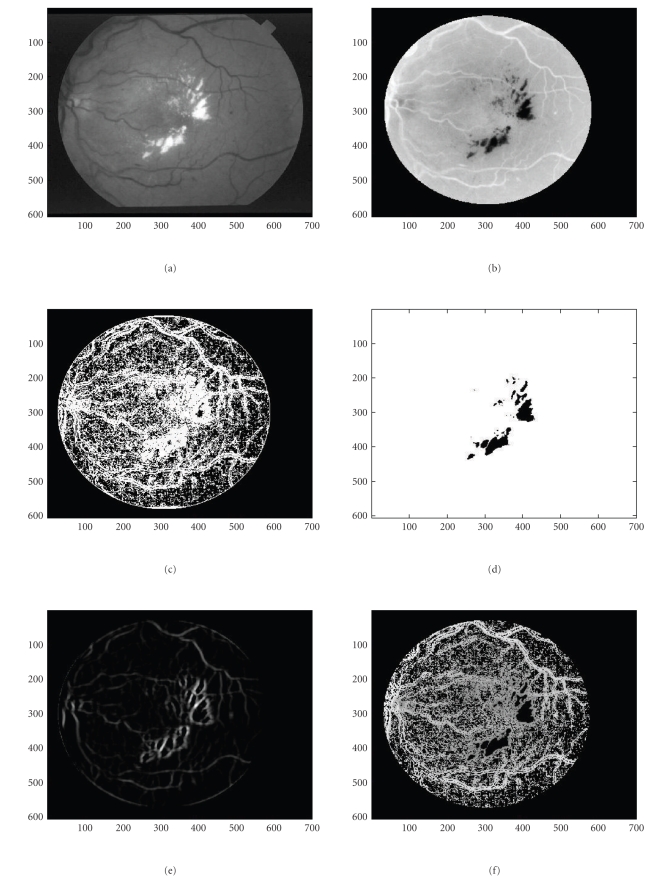

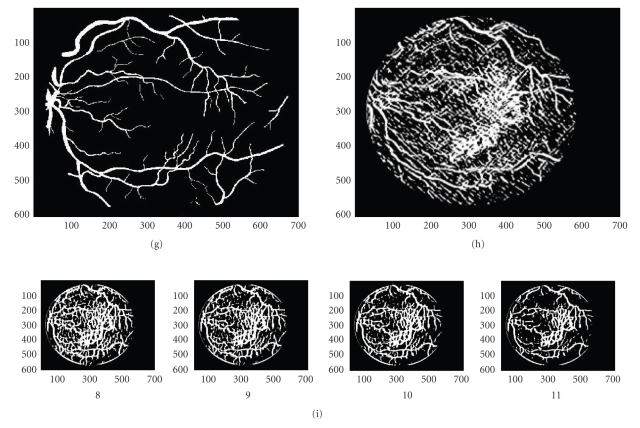
(a) Original fundus image for averagely traced major and peripheral network along with drusen. (b) Original fundus image ellipse corrected. (c) Binary vessel wall map. (d) Binary fundus image ellipse and drusen corrected. (e) Retinal vasculature with coarse and fine network based on VWP. (f) Retinal vasculature without VWP map using MIP. (g) Manually labelled ground truth vessel vasculature. (h) Redundant vessel vasculature produced using an augmented VM and double-sided GS-MG. (i) 2nd vessel network: Successive maps taken using different features and different sensitivities (*thresholds*). (8) Gradient homogeneity VMs. (9) Paired VP map and dominant gradient VM. (10) Increased vessel width allowed, lower gradient threshold and interpolated WPs. (11) Experimentally proved VM.

**Figure 4:**
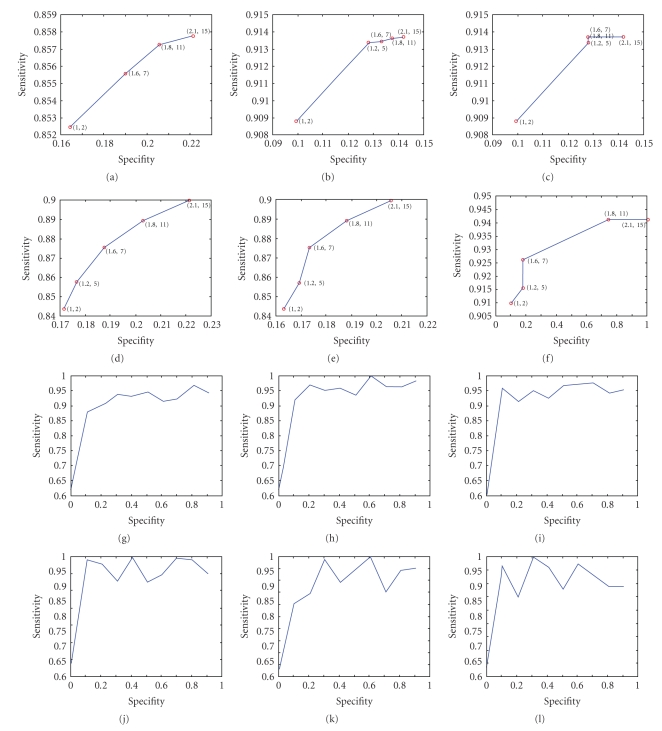
Comparative ROC curves between three full scans and three VWP-based scans: (a) case “2” vasculature with drusen, (b) for the more homogenous background vasculature in “1”, (c) extended drusen as in “3”. The homogenous vasculature in “b” gives a more straight relationship between *sensitivity *and *specificity* as a result of a uniform spatial distribution of FP's and TP's that does not allow relatively large variations as our threshold and VP pairing area radius increase. As for the guided scans the most notable observation is that SE is increasing less rapidly in the full scan case as observed in full scan graphs (d), (e) and (f) than in guided scan graphs (a), (b), and (c) as a result of faster TP detection offered a prior from VWP map. The Graphs (g), (h), and (i) show the results using double sided dual mode GS-MF on same vasculatures as those for which (a), (b), and (c) stand for. In Graphs (j), (k), and (l), ROC graphs are shown for the RG detection technique, the CL-based technique and three seed point technique for every branch of the vasculature greater in length than 10 pixels. All non VWM-guided techniques (g)⋯(l) have a steeper ROC for small specificity (SP) values. This shows when the number of FN is lowered the improvement/decrease of the FP is proportionally larger than with VWM guided detection. Our method directly addresses the FP decrease problem. Moreover, when we use more elaborated filters [spatially modulated dual mode double sided] the performance variation after the critical point where SE reaches a local peak (at SP≅.99, SE≅.1) is significantly lower than with RG, CL-based on 3 seed point.

**Figure 5:**
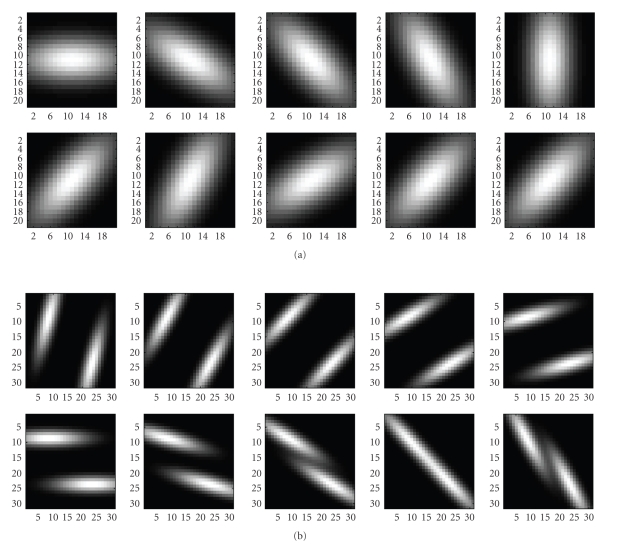
MF's used in different orientations and descending scales for fine VP detection: (a) single GS-MF. (b) double GS-MF with dual foreground and background transition modeling and indicative scale adjustment.

Some representative images are shown in (Figures 1–4)* where the asterisk “∗” indicates all subfigures or experiments referring to the same case. In (Figure 1)*, we show a not very clear background and a vessel network without any structural changes (drusen), with a very light background that does allow clearly seeing the peripheral vessel network details. In (Figure 3)* the vessel network has a more detailed peripheral vasculature and is sufficiently clear in that but still has some limited drusen, while in (Figure 2)* the background has a large amount of extended drusen. In (Figure 1)* and all cases, we show the original vessel network for all 3 vasculature types. Intermediate processing stages that are generated in the process are also shown in a figure series “∗” for all vasculature cases. These are produced by means of the wall detection mask and show the wall pixels, guiding the detection process. All stages are depicted with best candidate pixels. For visualization purposes wall pixels both give an idea of the basic network and also can alert on any evident missleading topologies like seriously broken segments or even abrupt cuts. Still, sparse pixels got separated using a finite, maximally 10-step region growing segmentation as in (Figure 5)* that lasted 10–15 seconds for the entire image. When a grown region recruited only a small number of pixels, typically (10≥15) then these pixels were set to the background class. The map methodology was examined in terms of time, false positives (FP's) and true positive (TP's) related ratios and all associated ROC analysis performers as will be explained later in this section. Pixel pairing, done with the use of a VWP map, removes significantly point characteristics very similar to vessel like point characteristics. The interior vessel-like network is thus limited in Figure 3(h) As it is shown in the case of (Figure 3)* the drussen have been algorithmically corrected. This was achieved by applying successive MFR threshold adaptation and local geometrical checking of the resulting pixels locations as described in the gradient coherence formula-criterion that is discussed in the Sections 2.2 and 4. We can then see the development of the results when spatial gradient coherence is applied as discussed where the spatial continuity of the gradient is imposed.

More specifically, in all three cases we present the original rough vessel pixel map, normalized in range [0,1] and polarity [light vessels, dark background]. The leading feature are the gradient features. When accounting for the entire set of features then the result is shown in the successive vasculature maps in Figure 3)* and on. When using the ordinary approach without the notion of the VWP map, then all the fundus pixels are considered. We get the vasculature network as shown in (Figure 4)* The standard MIP (*Maximum Intensity Projection*) algorithm is applied. We can clearly see that the vessel pixels are more diffused as in (Figures 3 and 4)* and there are many false positives that, even when using local segmentation, cannot be completely removed as they belong to vessel like neighborhoods. The results of successively adding more features to our algorithm can be studied in the individual feature maps as in (Figures 9(a)–9(c))*. In (Figure 8(a))* we see the application of a double-sided dual mode GS-MF Figure 5(b) as opposed to the single mode GS-MF (Figure 5(a)). These Figures depict a map of abundant vessel pixels with an increased number of FP's but not missed TP's. It is still similar to the simplest binary vasculature when only considering GL homogeneity, that is, when the local gradient is only considered and does not change too with regard to the original image but is being strong enough to give an evidence of local organized behaviour such as GL spatial differences. Furthermore, in (Figures 9(b))* more detailed vasculature maps can be observed when the likely pixels, which, in addition to possessing likely vessel-point properties can be also paired. Vessel pixel pairing reduces false alarms even more. In (Figures 9(c))* the straight and linear connectivity or linear continuum geometrical criterion is applied additionally. The ground truth vessel pixel maps are given in (Figure 7)* They are borrowed by the STARE and DRIVE databases [10, 13] where experts/ophthalmologists provide their insight on the vasculature they are presented with. If we optically inspect the original vasculatures with the GT as provided by the experts we can still see that faint [low contrast, narrow with] vessels are not perfectly captured. 

When applying local fine-segmentation the values for the thresholds for neighbors around likely vessel pixels depend on local circumstances. These can be spatial VWPs' GL standard deviation, or, the deviation of the distance between them. Still, the variation interval may lie within [1/2*μ*_*L*_, 2.5*μ*_*L*_], where

(7) $$\begin{array}{c} \mu_{L}\left(x_{o}\in R_{L}\right)=1/N_{L}*\left(\overset{}{\underset{\left(x,y\right)\in R_{L}}{\sum }}I\left(x,y\right)\right),\\ R_{L}\left({\overset{̅}{r}}_{o}\right)=\left\{r={\left(r_{x},r_{y}\right)}^{T}\exists ||\overset{̅}{r}-{\overset{̅}{r}}_{o}||\leq \text{STD}\left({\overset{̅}{r}}_{o}\right)\right\} \end{array}$$

is a local region around an examined seed-likely pixel (a VWP, i.e.), *μ*_*L*_ is the local GL average, $\text{STD}\left({\overset{̅}{r}}_{o}\right)$ is the local (centred at ${\overset{̅}{r}}_{o}$) GL's standard deviation. All these computations are preformed locally after the VWPs have been computed. Thus, they are very fast and computationally cheap. They are carried out only on selected regions around very likely vessel points. In fact, these fine local adjustments complete the effectiveness of more global descriptions, which are unable to capture very detailed local variations. They use parameters like convolution block size that are spatially varying. After most of the VWPs have been found the above local searches provide an updated version of the VPs, and improve the VWP map, they were based on. The final VPs account for the most part of the local information that is missed when applying more global criteria. The updated VWPs can be observed by comparing (Figure 3)* and (Figure 7) with (Figure 1)* and (Figure 3).

The resulting full vessel map is the result of inferring the interior VPs from the likely VWPs. The VWP approach offers also an insight into approaching vessel width. Nevertheless, fine vessel detection is a relativistic term. The major and peripheral networks entail parts of the fine vessel network. When the retina is highly contrasted and uniformly illuminated then a gradient map followed by a rectifying local spatial coherence adjustment can resolve the most important part of the network as in (Figure 3)*. When illumination is not uniform as in (Figure 1)* then local adaptation is needed to make clear details. 

**Figure 6:**
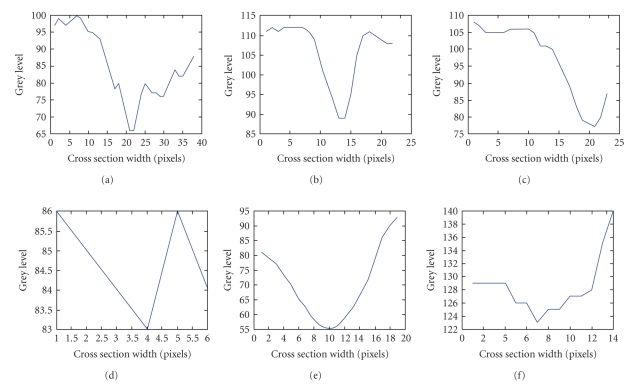
(a–f) Indicative cross section profiles encountered in 1D cross the line of CL: (a) mixed pattern that can be modelled both as double peaked and single peaked GS-MF for different peak values and spreads, (b,e) clear single peaked patterns, (c,f) single sided patterns modelling wall transition, (d) very shallow and narrow width vessel for pixel width.

**Figure 7:**
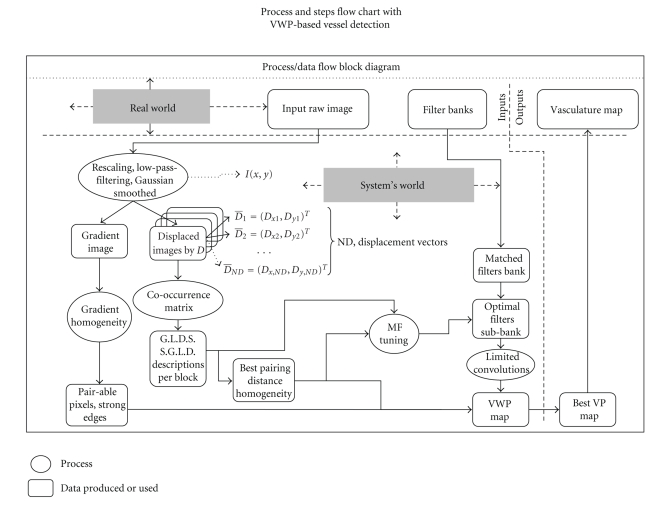
Process/Data Flow Chart depicting the basic information flow in our method.

Different features were used in increasing combinations as explained earlier in this section. The MFR gradient homogeneity offers strong vessel-like pixels but yields both vessel likely/false and true VPs. Among the high MFR gradient pixels those that can be paired for an acceptable range of vessel widths from 5 to 50 pixels consist a finer criterion applied. The width range applied offers increased width sensitivity and at the same time higher sensitivity/tolerance for roughly paired pixels. Expert proofed retinal images serving as a ground truth helped correcting local region-growing algorithms and thus to correct for the GL-MFR discontinuities. For high-resolution images of 4K × 4k the human eye sensitivity depends on the context or can be misled by noise. Sometimes the distinction between vessel walls and background even by human operators is not a trivial task. This can be proved by comparing different experts' opinions on retinal image details. This comparison is though beyond the scopes of the current Paper. In this case of confusing border pixels the only criterion that can be applied is the mathematical similarity to neighboring pixels. This solution is implemented by a few steps [3–10 iterations] region growing segmentation. The detection performance is directly connected to illumination modeling. We can use, though, established criteria that can help us to define performance. As already mentioned, this can be done using the ROC (*Receiver Operating Characteristic*) analysis on the results. Comparative ROC curves are given in Figures 6(a) and 6(c) for full scan. For VWP-guided scan, the results are more straight and similar looking since pixels are more accurately labelled. The ROC curves were tuned by major pixel pairing area and MFR thresholding parameters. They were both used in distinct parameter pairs (*r*_*L*_, th_MFR_), where (*r*_*L*_) is the maximally allowed local area's radius for paired pixels and (th_MFR_) is the linear proportion rate between acceptable MFRs and the mean value of the MFR field MFR_ACCEPTABLE_ > *t*h_MFR_ *μ*_MFR_. The two metrics used in all ROC curves were: x-axis: *sensitivity* (SE = TP/(TP + FN)), y-axis: *specificity *(SP = TN/(FP + TN)).

We can observe that full scan detection performs slightly worse since TP pixels are detected at higher rates as observed in the slope of (SE, SP(SE)) curve where SE(y-axis) is increasing versus SE's increments for nearly same SP's (x-axis). The amount of pixels examined are of the range of 4E+5 for a full scan while for a guided scan is of the range of less than 4.5E3 saving dramatically time. The accuracy is also better for a guided scan since high SE is achieved earlier.

The contribution of the present work is mainly in the lessening of the computational burden needed. For the nearly 100 times better scan-time we can use more advanced and sophisticated filtering. The method can serve thus as a step in removing many false alarms while missed TP or FN can be recovered near the area where TP's were initially observed. It is quite unlikely that major TP's are missed, by using a VWP, otherwise captured using a full scan, that is, without a wall map. One of the major features this work exploits is the pairing and symmetry as well as a colinearity of vessel pixels. Retinal pixels that cannot precisely fulfill these conditions and thus escape the two scans would probably be not observable even by experts. In this case the illumination modeling or the more effective noise removal can shed more light onto “hidden” TP's and can help thus bring to surface more FN's.

A representative accounting for compared processing times and ROC performance is given in Table 1. This is a composite performance indicator. This is the final detection time divided by the reliability of the system that is also referred to explicitly. The reliability is the area between the straight line (SE = SP) and the achieved line SE = *f*(SP), and is denoted as *D*[Area(SE)], where

(8) $$D\left(\text{Area}\left(\text{SP}\right)\right)=\overset{1}{\underset{0}{\int }}\text{SE}d\text{SP}-1/2*\;\text{SP}\left({\text{SE}}_{\text{SP}=0}-{\text{SE}}_{\text{SP}=1}\right).$$

We can observe that in all columns the reliability or the differential area is given as a separate denominator “/#” to show the range of performance between the methods compared. In columns “a” and “b” where the guided and full scan methods are compared, we had a relatively comparable differential area (quasi-equal denominators) while times differ by two scales of magnitude. This agrees with the rest of results where the trade-off between accuracy and speed is not lost in either favour [of speed or of accuracy] but also cannot be asserted or that the method trades speed for accuracy. In region growing (RG), as in column “d”, the times are in the range of a full scan vessel detection and this is expected since RG is not guided but saves some time from full scan in the sense that a user needs to elect good points as seed points that saves the system time. Part of this RG time is lost because of the relatively worse performance of RG due to less accurate and robust pixel inclusion criteria than those used in VWM pixel guided scan. With CL detection, as in column “c” the vessel is detected beginning from skeleton pixels. The performance denominator is significantly higher than in RG since the skeleton component of a vessel is half the way to have a successful vessel detection. Still, times are comparable with RG and full scan MF since even these skeleton points needs to be detected which takes time. The times for CL-based and RG were taken from own algorithms operated on same vessel data as VWM guided detection. Visual results are not provided due to space limitation.

In Table 2 the methods compared M1⋯M5 were reported in the literature both as working as stand-alone modules and also as separate modules in different systems. Some of the most common qualitative performance descriptions the authors were able to find in most works in the relevant literature are reported in Table 2. These refer to the automation degree a method offers, the accuracy it attains as well as the range of applications or its speciality and accuracy in vessel detection. Of special interest are vascular and neurovascular applications, which present different requirements in terms of the trade-off between accuracy and speed with respect to eye vessel detection. A CL-based method can be used in conjunction with an MF method in order to enhance local detection results. A CL in turn can be defined usually general purpose MFs that only detect the ridge of the CL but are unable to provide more insight into the local structure. There is thus no unanimously defined measure to compare arithmetically applications as the variety of applications diffuses specific modules performances. 

To more focus on our method reported as “M6” in Table 2 we can comment that it is not based on any selection of start or end point since the coarse as well as the fine detection are done automatically. It can be classified as suitable for tube-like structures since the use of advanced and complex MF serves primarily the purpose of accurately defining the diameter and the directional spread of the tubular bodies inside the vessel peripheral network. Hence, automation is a major advantage our method offers. Since not start or end points are needed no seed point is in question as perhaps RG methods or CL methods could use. It is most suitable for surgical planning and neurosurgical operations as it offers good accuracy and speed and none of them is obviously or proved traded over the other from our experiments. It cannot be used for detecting any kind of object as RG or CL methods could be more robust in. Still works on any GL—valued or colored image or any planar valued 2D or 3D field regardless the imaging modality that generated it. It applies both to main and peripheral vessel detection and depending on the choice of the MF parameters it works equally well as nonguided MF methods work given the area of application is known and the parameters are well selected. These two steps are inherent to our method as discussed but not in full scan, non-guided MF-based methods. 

The more quantitative indicators “gt”, “lt” do not refer to specific arithmetic evidences that can be safely found in the literature. They are derived as logical estimates from works in which the corresponding methods are referred to as individual steps to follow or as algorithms to run. Thus, they are mentioned as functional parts of larger processes, which classifies them as less accurate. or some methods usually are implemented as parts of others or super-parts of others. This justifies their quantitative relation “gt/lt” to them in terms or being of greater or lower efficiency in speed or accuracy.

## 6. Conclusions

In this paper, we presented a fully automated system for vessel detection on retinal images. The innovations that the current work clearly brings to the related research broadly relies on the way specific properties for the *Matched Filters *are dynamically computed using the core-notion of *Vessel Wall Pixels *(VWPs). This work makes extensive use of low-level, fast-to-compute features and advanced geometrical characteristics that are not often met in other related works. These properties are generally treated with intensive reasoning that exploits all aspects of the low-level visual information available. The texture information and the geometrical properties once computed are dynamically filtered by forcing homogeneity restrictions. Selected areas that exhibit high homogeneity in these features are used to fine-tune the MF's and restrict their parameters to their optimal values. This is not established theoretically though. We are inclined to think that complete theoretical treatment cannot be done to justify the best of choice as for the features we are using. We use ROC analysis as a classical semitheoretical performance indicator to make the point of the prons of our approach. We also consider the *time-to-detection *as a crucial performance indicator. The later is opposed to mainstream efforts in the area that rarely address the timing issue. We comment that sometimes the outperformance of our proposed schema is in trading performance for time. To this remark we would also add that the time could be a significant factor for a number of specific applications referring to the neurosurgery field as a potential example. 

We believe our paper raises a number of questions for discussion and further investigation. One is to better assess how to systematically tune optimal filters at the very local level when the application calls for it. Our approach suggests that the cocooccurrence matrix-based image's texture is a way to tackle with this issue. It is in our immediate plans to make the schema scale-independent by incorporating scale information into it. We are now working towards this direction. A second challenge to work on is the type of the trade-off that will be tolerated between processing complexity, processing time and performance. The last deals with the basic statement that not all image parts should be treated within the same computational framework and criteria must be established to trigger more advanced and computationally expensive modules. These modules should benefit from the computational power and the time that saved from the generic and lighter modules used in less interesting image parts.

## Figures and Tables

**Table 1:** Final detection time to reliability cost comparison between (A) full scan image detection w/o use of VM, (B) VM-guided scan, (C) CL-based vessel detection, and (D) Region growing (RG) with 3 points (start-intermediate-end) per vessel peripheral or main vessel segment (×10^2^/∗) (12 angles ∈[0,2*π*]/4 scales).

A	**B**	**C**	**D**
3,5860/.25	**0.0645/**.27	**2,930/0.1703**	**1.450/0.1025**
3.7373/.35	0.0678/0.091	1.8686/0.2210	.525/0.021
4.2423/.37	0.0327/0.3821	2.100/0.1345	.789/0.1177
**2.2557/.27**	**0.0671/0.1486**	**1.120/0.2442**	**.678/**0.0884
**4.0721/0.3950**	**0.0345/0.2799**	**2.030/0.2317**	**1.789/0.1322**
**2.2868/0.3021**	**0.0893/0.0883**	**1.100/0.1046**	**456/0.0865**
**3.4912/0.2896**	**0.0569/0.3204**	**1.740/0.1192**	**1.679/0.1310**
2,5870/0.3971	**0.0895/0.2082**	**2.835/0.2143**	**1.456/0.0616**

**Table 2:** Comparative analysis of the main vessel detection techniques with the proposed one.

Methodology Followed	Start/End Point Needed	Tube Specific	No Automation	Seed Point Needed	time to detection	Surgical planning	Neuro-surgical planning	Arbitrary objects detection	Arbitrary image modality	Fine Vessel Accuracy	Main vessel Accuracy	Center-Line-Detection Accuracy	Module complexity	Feature Complexity
m1	y	n	y	y	gt	Y	Y	n	y	gt	lt	y	gt	Lt
m2	y	y	y	n	gt	Y	y	y	y	gt	gt	lt	lt	eq
m3	y	y	n	n	gt	n	n	y	y	lt	eq	eq	lt	lt
m4	y	n	n	n	lt	y	y	y	y	lt	lt	eq	lt	lt
m5	y	n	y	y	gt	n	n	y	y	lt	y	lt	lt	lt
m6	n	y	n	n	eq	y	y	n	y	eq	eq	eq	eq	eq

**Table 3:** Designations for methods or sub-modules used in Table 2.

M1	*Multiple seed point*	GT	*Designates that examined descriptor is higher in this method than with our method*
M2	*MF (full scan)*	LT	*Designates that examined descriptor is lower using this method than using ours *
M3	*CL detection*	Eq	*The examined and our method are roughly equal in that feature*
M4	*Start-End point per vessel segment*	Y	*Holds most likely true for both our and examined method*
M5	*Region Growing*	N	*Does not hold true for both our and examined method*
M6	*Proposed*		
